# Canakinumab treatment patterns in sJIA, FMF, TRAPS, and MKD/HIDS: real-world insights from a Belgian non-interventional study

**DOI:** 10.1186/s41927-025-00515-w

**Published:** 2025-05-29

**Authors:** Michel Moutschen, Cécile Boulanger, Joke Dehoorne, Rik Joos, Florence Roufosse, Vito Sabato, Jeroen van der Hilst, Eleonore Maury, Hilde Rabijns, Marijn Witterzeel, Carine Wouters

**Affiliations:** 1 Clinical Immunology and Internal Medicine, CHU de Liège, ULiège, Liège, Belgium; 2 Department of Pediatric Hematology and Oncology, Cliniques Universitaires Saint-Luc, Brussels, Belgium; 3 Department of Pediatric Rheumatology, Ghent University Hospital, Ghent, Belgium; 4 European Reference Network for Rare Immunodeficiency, Autoinflammatory and Autoimmune Diseases, Ghent University Hospital, Ghent, Belgium; 5 Department of Rheumatology, ZNA Jan Palfijn, Antwerp, Belgium; 6 Department of Internal Medicine, Hôpital Universitaire de Bruxelles– Erasme, Université Libre de Bruxelles, Brussels, Belgium; 7 Department of Immunology, Allergology, Rheumatology and the Infla-Med Centre of Excellence, Faculty of Medicine and Health Sciences, University of Antwerp, Antwerp, Belgium; 8 Limburg Clinical Research Center, Hasselt University, Hasselt, Belgium; 9 Department of Infectious Diseases and Immune Pathology, Jessa General Hospital, Hasselt, Belgium; 10 Novartis Pharma NV/SA, Vilvoorde, Belgium; 11 Division of Pediatric Rheumatology, Department of Pediatrics, University Hospitals Leuven, Leuven, Belgium

**Keywords:** Autoinflammatory diseases, Canakinumab, Familial Mediterranean fever (FMF), Hyperimmunoglobulinemia D syndrome (HIDS), Mevalonate kinase deficiency (MKD), Systemic juvenile idiopathic arthritis (sJIA), Tumor necrosis factor receptor-associated periodic syndrome (TRAPS)

## Abstract

**Background:**

Canakinumab, an IL-1β inhibitor, has demonstrated long-term efficacy and safety in patients with sJIA, FMF, TRAPS, and MKD/HIDS who experience inadequate disease control with conventional treatments. This non-interventional study aimed to gain insights into canakinumab use and treatment patterns for these diseases in Belgium.

**Methods:**

Between July 1, 2018 and June 30, 2023, this national, non-interventional, retrospective/prospective study enrolled patients aged ≥ 2 years with sJIA, FMF, TRAPS, or MKD/HIDS reimbursed for, and treated with, canakinumab in Belgium. Part 1: retrospective data collection from first canakinumab administration in the initial 6-month reimbursement period until date of study inclusion. Part 2: prospective data collection following study inclusion. Canakinumab treatment and safety data were collected throughout.

**Results:**

At data cut-off, 96 patients (7 sJIA, 70 FMF, 13 TRAPS, 6 MKD/HIDS) were enrolled, of whom 54.2% were female and 87.5% were adults (aged ≥ 18 years). Median age at first canakinumab administration was 34.0 years (20.0, 35.0, 37.0, and 42.0 years in sJIA, FMF, TRAPS, and MKD/HIDS, respectively). Eighteen patients discontinued treatment (3 sJIA, 11 FMF, 4 TRAPS), which was due to lack of efficacy (per investigator’s judgment) in 10 (10.4%) patients. Median dose per administration was 289.1 mg in patients with sJIA, and 150.0 mg in patients with FMF, TRAPS, and MKD/HIDS, while median interval between two consecutive administrations was 28.0 days. Thirty-five (36.5%) patients with FMF, TRAPS, or MKD/HIDS received ≥ 1 dose increase (≥ 150 mg). No safety events were reported.

**Conclusions:**

These non-interventional study data highlight that canakinumab treatment patterns are generally aligned with the summary of product characteristics (SmPC) and reimbursement criteria in Belgium and further support the well-tolerated safety profile of canakinumab. However, Belgian reimbursement criteria require long-term glucocorticoids prior to canakinumab therapy; if it were possible to align treatment more closely with EULAR/PReS guidance, which recommends early initiation of anti-IL-1 or anti-IL-6 therapy, glucocorticoid treatment would be limited and improved outcomes for these patients would likely be possible.

**Clinical trial number:**

Not applicable.

**Supplementary Information:**

The online version contains supplementary material available at 10.1186/s41927-025-00515-w.

## Background

Familial Mediterranean fever (FMF), tumor-necrosis factor receptor-associated periodic syndrome (TRAPS) and mevalonate-kinase deficiency (MKD), also known as hyperimmunoglobulin-D syndrome (HIDS), are a group of rare monogenic autoinflammatory diseases (AID) driven by the pro-inflammatory cytokine interleukin (IL)-1 [1]. Still’s disease, sometimes subcategorized into systemic juvenile idiopathic arthritis (sJIA) and adult-onset Still’s disease (AOSD), is a rare polygenic AID that shares pathophysiologic mechanisms with monogenic AIDs [1].

These disorders are most often characterized by unprovoked and recurrent episodes of fever lasting several days or weeks, serositis, arthritis, and/or systemic inflammation, depending on the individual condition [2–4]. In the absence of adequate disease control, patients with AIDs are at risk of developing serious and potentially irreversible conditions related to persistent systemic inflammation, including joint or neurological damage, osteoporosis, growth impairment, and amyloidosis (specifically renal amyloidosis, which may result in renal failure) [3, 5–7]. In addition to an increased risk of progression to serious complications, AIDs can substantially impact a patient’s long-term health-related quality of life (HRQoL) through the unpredictable and frequent nature of disease flares [8,9].

According to treatment recommendations and guidelines, the ultimate goals in the management of AIDs relate to control of systemic inflammation [4, 10, 11]. Conventional treatments for these diseases include non-steroidal anti-inflammatory drugs (NSAIDs) and glucocorticoids for temporary symptom relief during flares; however, these treatments fail to control disease activity in some patients, and their long-term use is associated with systemic side effects [4, 10, 11]. Colchicine is the mainstay of FMF treatment, although some patients experience colchicine resistance or intolerance (approximately 5–10% and up to 20%, respectively), leading to inadequate disease control and an increased risk of persistent inflammation [10, 12–15]. Due to the role of pro-inflammatory cytokines in the pathogenesis of AIDs, targeted biologic therapy, such as anti-IL-1 treatment, is an effective option for patients experiencing inadequate disease control with conventional treatments [16, 17]. Guidelines from the European Alliance of Associations of Rheumatology (EULAR), Pediatric Rheumatology European Society (PReS), and American College of Rheumatology recommend the initiation of biologics as early as possible after a diagnosis of sJIA, TRAPS, or MKD/HIDS [4, 11]. In FMF, supplemental treatment with biologics is indicated in patients experiencing persistent flares or inflammation who are inadequately controlled at the maximum tolerated dose of colchicine [10].

Canakinumab is a fully human, monoclonal antibody that selectively binds to IL-1β, blocking signaling through its receptor and disrupting the inflammation pathway [18, 19]. It is approved by the European Medicines Agency and the United States Food and Drug Administration for the treatment of Still’s disease, gouty arthritis/gout flares, cryopyrin-associated periodic syndromes (CAPS), FMF, TRAPS, and MKD/HIDS [18, 19]. In patients with sJIA, the recommended starting dose (RSD) is 4 mg/kg (up to a maximum of 300 mg) subcutaneously every 4 weeks in patients aged ≥ 2 years and weighing ≥ 7.5 kg. In patients with FMF, TRAPS, and MKD/HIDS, the RSD is 150 mg (or 2 mg/kg for patients weighing ≥ 7.5 kg and ≤ 40 kg) subcutaneously every 4 weeks in patients aged ≥ 2 years and weighing ≥ 7.5 kg [18]. An intensified canakinumab dose (up to 300 mg, or 4 mg/kg in patients weighing ≤ 40 kg) is recommended in patients with FMF, TRAPS, and MKD/HIDS who have had an inadequate clinical response to the RSD [18]. Long-term efficacy, alongside a well-tolerated safety profile, has been demonstrated with canakinumab in the treatment of AIDs in several phase 3 clinical trials [20–22].

Real-world evidence (RWE) can also provide valuable insights into the treatment patterns and management of these diseases, where the rare nature of these conditions limits recruitment in clinical trials [23]. In an analysis of clinical practice data from the German AID-registry, canakinumab was well tolerated in patients with sJIA, and 85% of treated patients achieved inactive disease within 1 year [24]. In addition, an analysis of the Juvenile Inflammatory Rheumatism (JIR) cohort (an international data repository of juvenile-onset AIDs) showed that physicians routinely adjust the canakinumab dosage in patients with FMF, TRAPS, and MKD/HIDS, and the optimal dosage required to control disease activity varies by indication [25, 26]. Recently, results from an analysis of the RELIANCE non-interventional study confirmed the long-term safety profile of canakinumab and its ability to effectively control disease activity in patients with FMF, TRAPS, and MKD/HIDS, additionally highlighting the impact of treat-to-target strategies on achieving improved disease control and HRQoL in real-world settings [27].

This non-interventional study was conducted in response to a request from the Belgian health authorities to gain insights into the use and treatment patterns of canakinumab in patients with sJIA, FMF, TRAPS, and MKD/HIDS who received reimbursement for, and were treated with, this biologic in Belgium during the study period. The availability of these non-interventional study data will further strengthen current understanding and, when considered with other RWE, could help inform clinical practice and management in these patient populations.

## Methods

### Study design and patients

This non-interventional study is a national, retrospective/prospective study evaluating canakinumab treatment patterns in patients with sJIA, FMF, TRAPS, or MKD/HIDS from nine study centers in Belgium. No patients with AOSD or CAPS were enrolled as these indications were not subject to the request from the Belgian health authorities.

To receive canakinumab in Belgium, patients must meet all reimbursement criteria and there are no alternative methods to receive funding; full reimbursement criteria can be found on the National Institute for Health and Disability Insurance (RIZIV/INAMI) website and criteria from the time of data collection can be found in Additional file 1: Suppl. Table 1 [28]. Key criteria include: a preceding course of glucocorticoids (3–6 months, dependent on dosing) and failure with the IL-6 inhibitor tocilizumab prior to canakinumab initiation in patients with insufficiently controlled sJIA or documented active FMF (1 attack per month over the past 12 months, despite maximal tolerated colchicine dosing) or active TRAPS or MKD/HIDS (1 attack per month over the past 12 and 6 months, respectively). There were two reimbursement periods in this study, in line with the reimbursement criteria: an initial 6-month period, and consecutive 12-month prolongation periods for patients who responded to canakinumab during the first 6 months.

**Fig. 1:**
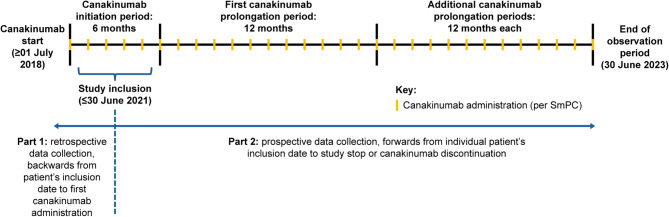
Study design. *Abbreviations*: SmPC, summary of product characteristics

This study comprised two parts: Part 1 included the retrospective collection of data from the first canakinumab administration in the initial 6-month reimbursement period until the date of inclusion in the study, while Part 2 included the prospective collection of data following inclusion in the study for each patient (Fig. 1). Patients were included in the study upon receipt of written informed consent, while the observation period for each patient was the date of first canakinumab administration until treatment discontinuation or end of study, whichever occurred first. For this reason, the non-interventional study inclusion date could be later than the start of the observation period and initiation of canakinumab treatment.

The study started on July 1, 2018, with an enrollment period of 3 years (until June 30, 2021) and a maximum observation period of 5 years; the cut-off date for this final analysis was June 30, 2023. Canakinumab treatment was initiated by the treating physician according to standard of care and local clinical practice, in line with the summary of product characteristics (SmPC), the medical needs of the patient, and the reimbursement criteria [18, 28]. No predefined criteria for treatment response, lack of efficacy, and remission were stipulated due to the observational nature of the study; these events were determined per investigators’ judgment. However, canakinumab was administered in line with reimbursement criteria, which stipulate that treatment cannot continue if response is not achieved (Additional file 1: Suppl. Table 1) [28]. Reimbursement criteria define response as follows: sJIA patients must achieve an ACR Pedi 30 response compared with their clinical condition before treatment, and absence of fever associated with sJIA (no temperature ≥ 37.5 °C in the previous 7 days) after 6 months and every 12 months thereafter; patients with FMF, HIDS or TRAPS must achieve CRP < 10 mg/l and/or > 70% reduction from baseline, as well as a PGA score < 2, after 16 weeks and every 12 months thereafter [28].

Eligible patients were those with sJIA, FMF, TRAPS, or MKD/HIDS (aged ≥ 2 years) who were granted reimbursement for, and were treated with, canakinumab, and provided written informed consent (provided by the patient’s parent or legal representative, where appropriate). Patients were excluded from the study solely if they did not fulfil all the inclusion criteria, no other reasons for exclusion were applied.

This study was conducted in accordance with the principles of the Declaration of Helsinki. All participating centers obtained approval from their local, non-leading ethics committees, and the leading ethics committee (Independent Ethics Committee of the CHU de Liège) approved the protocol.

### Endpoints

The primary endpoint was the dosage of canakinumab given at each administration during the observation period, per patient. Secondary endpoints included: the number of patients who were granted reimbursement for, and received, canakinumab treatment during the inclusion and observation periods; age at first canakinumab administration; the number of patients who discontinued canakinumab and the reasons for discontinuation; body weight at baseline and at each canakinumab administration during the observation period; and safety events experienced during the observation period.

### Data collection

For each patient, data were collected retrospectively (at study inclusion) and prospectively (upon every subsequent canakinumab administration). Patient-level data regarding canakinumab treatment (dosage, discontinuation), body weight, and safety measures (adverse events [AEs], serious AEs [SAEs], adverse drug reactions [ADRs], and serious ADRs [SADRs]) were collected throughout the study; data were subsequently deidentified and aggregated. The study database was reconciled following study completion.

### Statistical analysis

Data were reported descriptively and no statistical hypothesis testing was performed. In addition, no sensitivity analyses were identified in the statistical analysis plan. Only data collected during routine clinical practice were included in the non-interventional study; for this reason, some missing data were anticipated. No substitution, imputation, or other correction methods were used to complete missing data, as the occurrence could not be presumed to be random.

## Results

### Demographics and disease characteristics

#### Demographics

Data are available for a total of 96 patients (7 sJIA, 70 FMF, 13 TRAPS, 6 MKD/HIDS) enrolled between July 1, 2018 and June 30, 2021 (Table 1). At baseline, 84 (87.5%) patients were aged ≥ 18 years; 52 (54.2%) patients were female. Approximately one-third (32.3%) of patients weighed ≥ 40 kg; body weight data were missing for 60 (62.5%) patients.

**Table 1:** Demographics in the pooled population

	All patients (*N* = 96)
Diagnosis, *n* (%)	
sJIA	7 (7.3)
FMF	70 (72.9)
TRAPS	13 (13.5)
MKD/HIDS	6 (6.3)
Age, years	
Median (range)	34.0 (3.0–67.0)
* Age groups*,* n (%)*	
< 12 years	4 (4.2)
12–17 years	8 (8.3)
≥ 18 years	84 (87.5)
Sex, *n* (%)	
Female	52 (54.2)
Body weight at initiation, *n* (%)	
< 40 kg	5 (5.2)
≥ 40 kg	31 (32.3)
Missing	60 (62.5)

Abbreviations: FMF: familial Mediterranean fever; MKD/HIDS: mevalonate kinase deficiency/hyperimmunoglobulin-D syndrome; sJIA, systemic juvenile idiopathic arthritis; TRAPS: tumor necrosis factor receptor-associated periodic syndrome

#### Age at first canakinumab administration

**Fig. 2:**
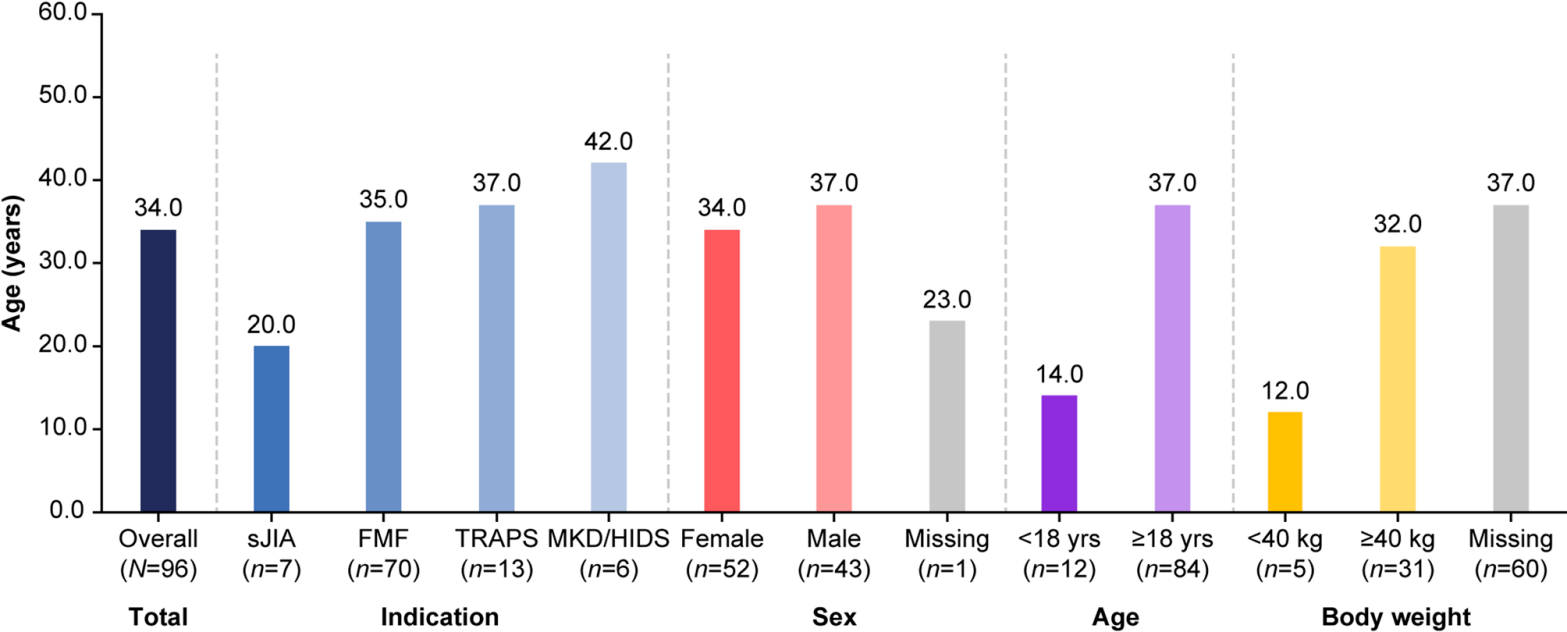
Median age at first administration of canakinumab per indication, sex, age group, and body weight. *Abbreviations*: FMF: familial Mediterranean fever; MKD/HIDS: mevalonate kinase deficiency/hyperimmunoglobulin-D syndrome; sJIA: systemic juvenile idiopathic arthritis; TRAPS: tumor necrosis factor receptor-associated periodic syndrome

The median (range) age at first canakinumab administration was 34.0 (3.0–67.0) years (Fig. 2). The median (range) age at first administration was 20.0 (16.0–35.0) years in patients with sJIA, 35.0 (8.0–65.0) years for FMF, 37.0 (3.0–67.0) years for TRAPS, and 42.0 (26.0–51.0) years for MKD/HIDS. In patients aged < 18 years and ≥ 18 years, the median (range) age at first administration was 14.0 (3.0–16.0) and 37.0 (18.0–67.0) years, respectively.

### Treatment duration

The maximum observation period for each patient was 60 months; due to the study design, the observation period was equal to the duration of canakinumab treatment received during the study. The median (range) duration of canakinumab treatment was 44.8 (0.0–59.4) months; 1 patient received one dose only (duration 0 months). By indication, the median (range) duration of treatment was 49.5 (2.4–59.3) months in sJIA, 47.4 (0.0–59.4) months in FMF, 29.9 (12.7–52.5) months in TRAPS, and 42.6 (12.7–49.2) months in MKD/HIDS.

### Treatment discontinuation

**Fig. 3:**
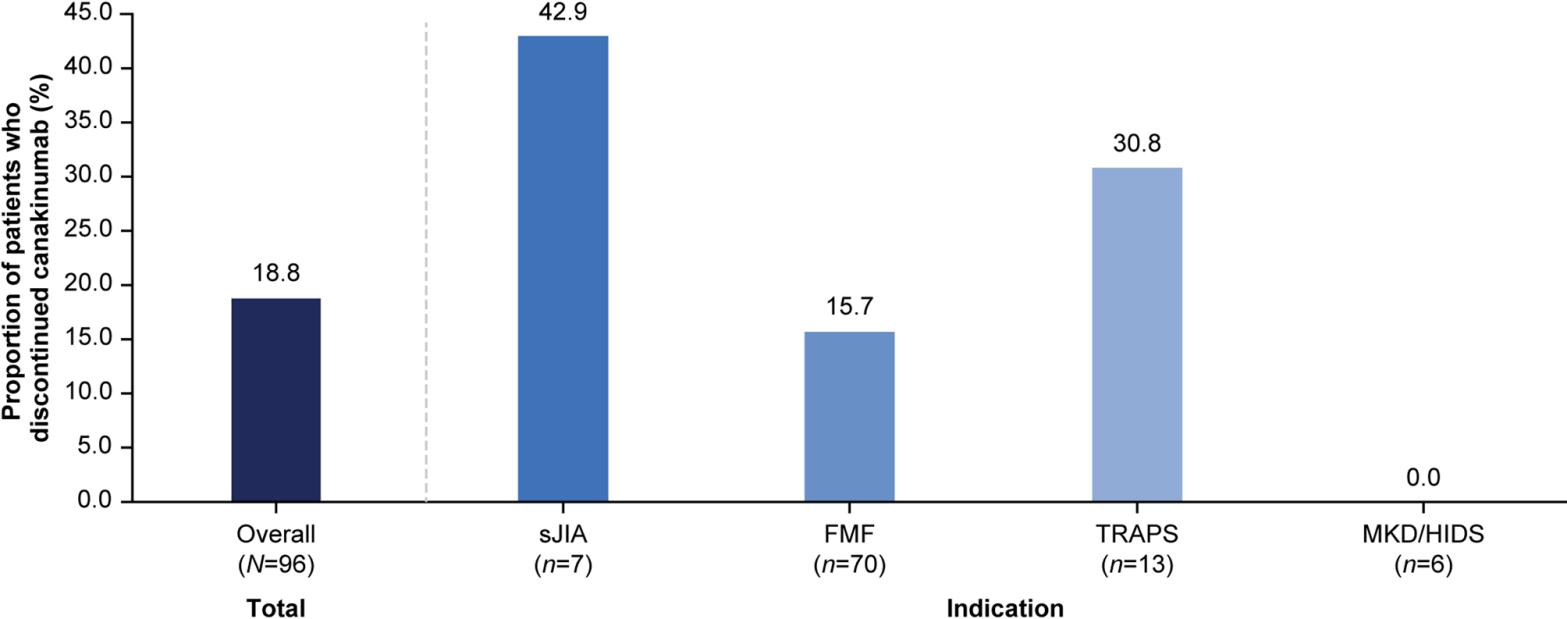
Canakinumab discontinuations during the study. *Abbreviations*: FMF: familial Mediterranean fever; MKD/HIDS: mevalonate kinase deficiency/hyperimmunoglobulin-D syndrome; sJIA: systemic juvenile idiopathic arthritis; TRAPS: tumor necrosis factor receptor-associated periodic syndrome

Overall, 18 (18.8%) patients discontinued canakinumab treatment during the observation period (Fig. 3), including 3 patients with sJIA (42.9% of the sJIA cohort), 11 with FMF (15.7% of the FMF cohort), and 4 with TRAPS (30.8% of the TRAPS cohort). No patient with MKD/HIDS discontinued treatment during the study. In only one center, there were no treatment discontinuations; in the eight remaining centers, discontinuation was evenly distributed, with at least one discontinuation in each. Reasons for discontinuation included lack of efficacy (10.4%: all 3 with sJIA, 6 FMF, 1 TRAPS) and remission (2.1%: 1 FMF, 1 TRAPS), per the investigators’ judgment. In addition, some patients were lost to follow-up (*n* = 2), or discontinued treatment due to “inadequate treatment”, pregnancy wish, relocation abroad, and unknown (*n* = 1 each). For the patients who discontinued due to lack of efficacy, the median (range) number of canakinumab administrations was 5.5 (1.0–30.0); per indication, this was 5.0 (4.0–13.0) in patients with sJIA, 5.5 (1.0–30.0) in patients with FMF, and 14.0 (14.0–14.0) in the patient with TRAPS.

Overall, most patients (*n* = 78; 81.3%) were still receiving canakinumab at the end of the observation period. In these patients, the median (range) duration of treatment was 48.5 (8.8–59.4) months. By indication, the median (range) duration of treatment when excluding discontinuations was 55.2 (49.5–59.3) months in sJIA (*n* = 4), 49.7 (8.8–59.4) months in FMF (*n* = 59), and 30.3 (23.9–52.5) months in TRAPS (*n* = 9).

### Canakinumab dosing

**Fig. 4:**
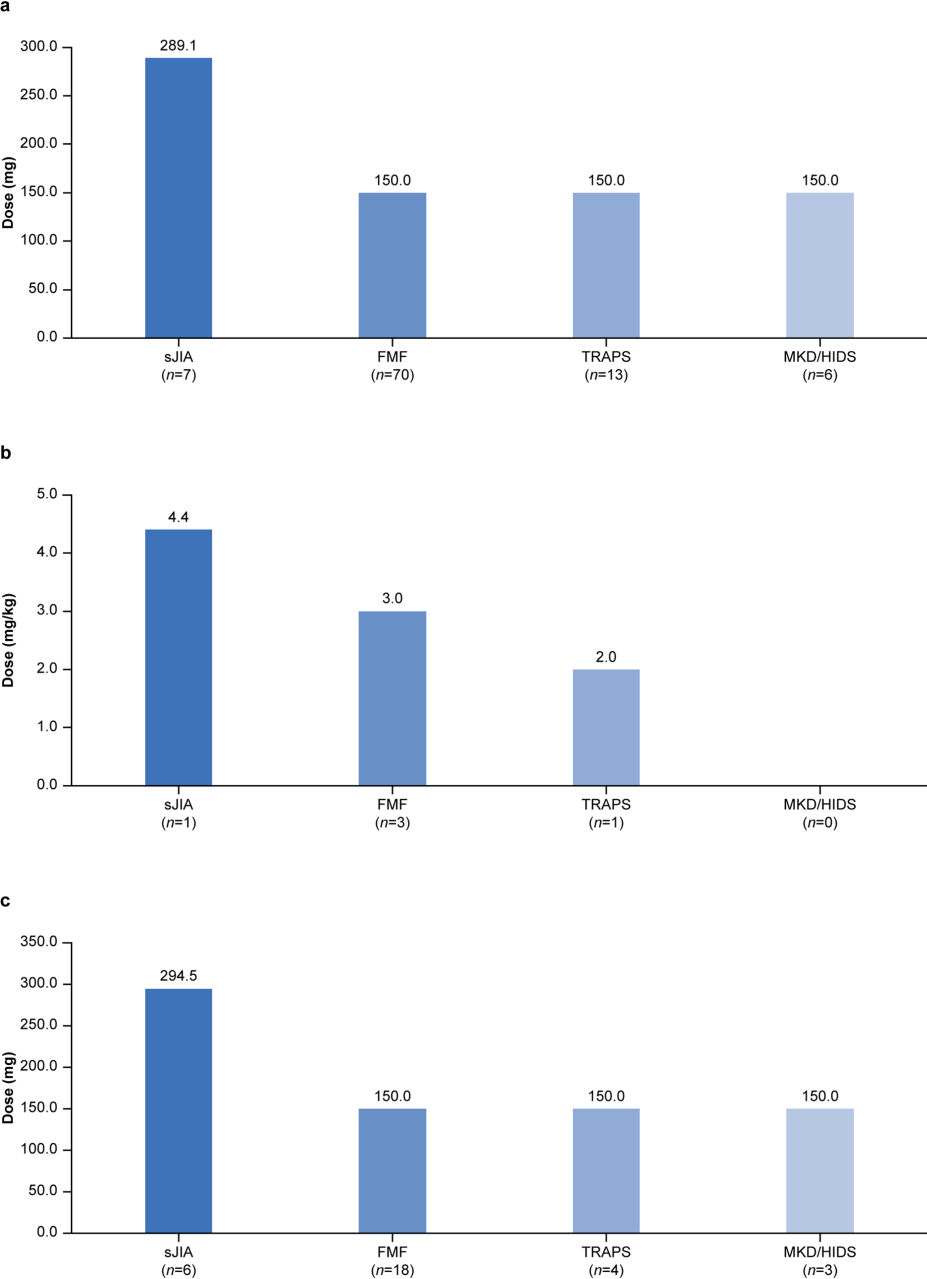
Median dose per administration per indication in the total population (**a**), patients weighing < 40 kg (**b**), and patients weighing ≥ 40 kg (**c**). *Abbreviations*: FMF: familial Mediterranean fever; MKD/HIDS: mevalonate kinase deficiency/hyperimmunoglobulin-D syndrome; sJIA: systemic juvenile idiopathic arthritis; TRAPS: tumor necrosis factor receptor-associated periodic syndrome

By indication, the median (range) dosing was 289.1 mg (150.0–447.6 mg) in patients with sJIA, 150.0 mg (101.6–281.6 mg) in patients with FMF, 150.0 mg (33.3–276.9 mg) in patients with TRAPS, and 150.0 mg (150.0–265.4 mg) in patients with MKD/HIDS (Fig. 4). Overall, 35 patients received ≥ 1 dose increase (≥ 150 mg), with the higher dose maintained until study end in 8 patients (Table 2). Per indication, 28 (40.0%) patients with FMF, 6 (46.2%) with TRAPS, and 1 (16.7%) with MKD/HIDS received ≥ 1 dose increase; patients with sJIA were excluded due to the higher RSD in these patients. There were ≥ 1 off-label dose increases (> 300 mg) in 5 patients with FMF and none in patients with TRAPS or MKD/HIDS. There were no dose reductions (< 150 mg) in patients weighing ≥ 40 kg; patients weighing < 40 kg were excluded from this analysis as they received weight-based dosing. The mean dosing per indication in patients weighing < 40 kg and ≥ 40 kg is shown in Fig. 4. As body weight data were missing for 62.5% of patients, change in dosing relative to body weight changes could not be analyzed.

**Table 2:** Canakinumab dose increases during the study in patients with FMF, TRAPS, and MKD/HIDS

		Indication		
	Overall	FMF	TRAPS	MKD/HIDS
Total number of patients, n*	77	60	11	6
Patients receiving ≥ 1 dose increase during the study, *n* (%)^†^	35 (45.5)	28 (46.7)	6 (54.5)	1 (16.7)
Patients receiving increased dose until the end of the study, *n* (%)^‡^	8 (22.9)	5 (17.9)	2 (33.3)	1 (100.0)

*Patients aged <18 years, and those who discontinued canakinumab treatment after one administration, were excluded

†Dose increase of ≥150 mg

‡Independent of the number of increased doses received during the study

Abbreviations: FMF: familial Mediterranean fever; MKD/HIDS: mevalonate kinase deficiency/hyperimmunoglobulin-D syndrome; TRAPS: tumor necrosis factor receptor-associated periodic syndrome

### Interval between canakinumab administrations

A total of 3259 intervals between two consecutive administrations were recorded; per indication, 252 intervals in sJIA, 2472 intervals in FMF, 367 intervals in TRAPS, and 168 intervals in MKD/HIDS were recorded. The median (range) interval between two consecutive administrations was 28.0 (1.0–691.0) days. By indication, the median (range) interval between administrations was 28.0 (3.0–56.0) days in sJIA, 28.0 (1.0–654.0) days in FMF, 28.0 (2.0–406.0) days in TRAPS, and 28.0 (7.0–691.0) days in MKD/HIDS. Patients receiving only one canakinumab administration during the study (*n* = 2, both FMF) were excluded from this subanalysis.

### Safety

No AEs, SAEs, ADRs, or SADRs were reported by investigators in the case report forms of this non-interventional study. As detailed above, 10 (10.4%) patients discontinued canakinumab treatment due to a lack of efficacy during the observation period, which were later reported as AEs during the reconciliation of the study database by Novartis.

## Discussion

The present study is one of the largest datasets describing long-term canakinumab treatment patterns in patients with sJIA, FMF, TRAPS, and MKD/HIDS. Overall, the findings from this dataset indicate that real-world treatment patterns for most patients in Belgium are generally aligned with the SmPC and the reimbursement criteria, and further demonstrate the well-tolerated safety profile of canakinumab.

For patients in the study, canakinumab was generally initiated at a notably older age compared with pivotal trial data in patients with sJIA (median 20.0 vs. 8.0 years, respectively), FMF (median 35.0 vs. mean 22.5 years, respectively), TRAPS (median 37.0 vs. mean 21.0 years, respectively), and MKD/HIDS (median 42.0 vs. mean 13.0 years, respectively) [20, 21]. The ages at canakinumab initiation reported here are also higher than those seen in other RWE, which were median 8.7 years, 14.4 years, 18.9 years, and 9.7 years for sJIA, FMF, TRAPS, and MKD/HIDS, respectively [24, 26]. In addition, median ages at inclusion in the RELIANCE FMF, TRAPS, and MKD/HIDS cohorts (23.0, 23.0, and 7.0 years, respectively) were lower than those observed in our study, yet canakinumab was initiated prior to the start of the RELIANCE non-interventional study in most patients [27].

The delay in canakinumab initiation likely relates to the lack of canakinumab access before reimbursement (prior to July 1, 2018), and the restricted criteria thereafter. Particularly, per the European label for sJIA, canakinumab is indicated in patients who have responded inadequately to previous therapy with NSAIDs and systemic glucocorticoids [18]. However, per the Belgian reimbursement criteria, patients with sJIA must have failed to respond to treatment with tocilizumab, in addition to long-term use of glucocorticoids (3–6 months), prior to canakinumab initiation [28]. This is not in agreement with the recently published EULAR/PReS recommendations, nor the SmPC [11, 18]. Specifically, EULAR/PreS recommendations advise that long-term use of glucocorticoids to achieve and maintain treatment goals must be avoided, and highlight that treatment with IL-1 or IL-6 inhibitors should be initiated as early as possible following diagnosis [11]. Furthermore, it is increasingly recognized that, due to the biphasic nature of sJIA, a potential treatment window of opportunity exists, where early initiation of IL-1 inhibitors in the systemic phase may prevent disease progression and its complications in some patients [29, 30]. There is a discrepancy between this concept and the mandatory long-term use of glucocorticoids in patients with sJIA in Belgium. Alternate anti-IL-1 treatment with anakinra is reimbursed in Belgium for the management of sJIA following the same criteria, although only since March 2019. Previous exposure to biologics was not evaluated in this study, but as the canakinumab reimbursement criteria stipulate failure with tocilizumab treatment (Additional file 1: Suppl. Table 1), previous exposure to tocilizumab is almost certain. Pre-treatment with anakinra is however unlikely for 5 out of 7 sJIA patients in this study, as they were already receiving canakinumab by the time anakinra was reimbursed. In FMF, anakinra only obtained reimbursement in September 2021 [30] which was after the stop date of inclusion in this study and for TRAPS and HIDS, anakinra is not EMA approved; tocilizumab is not approved in these three indications. As the Belgian reimbursement system dictates access, the likelihood of biological pre-treatment in these indications is very low.

The median treatment duration was 44.8 months (48.5 months when excluding discontinuations) in this study; 18.8% of patients discontinued canakinumab, including 10.4% due to lack of efficacy. Of 18 patients who discontinued canakinumab, 11 did so within the initial 6-month reimbursement period, suggesting that physicians did not continue canakinumab treatment if it was not suitable for the patient. Of the patients with sJIA who discontinued treatment, all 3 did so due to lack of efficacy (42.9% of the sJIA cohort), which is substantially higher than was seen in the German AID-registry (3.7%), and in clinical trials assessing canakinumab in patients with sJIA (10.5–14.7%) [22, 24, 31]. This suggests that patients with sJIA included in this study were a refractory population; this could be expected since, as previously discussed, the Belgian reimbursement criteria limit access only to patients who have failed to respond to long-term glucocorticoids and tocilizumab treatment (Additional file 1: Suppl. Table 1) [28]. Conversely, canakinumab is initiated per physician judgment in Germany and reimbursement follows this decision, rather than specific reimbursement criteria; due to this, a broader population may be included in the German AID-registry and other German registries. In addition, classification criteria for the diagnosis of sJIA are less stringent than those for FMF, TRAPS, and MKD/HIDS, where the roles of *MEFV*, *TNFRSF1A*, and *MVK* mutations, respectively, are well established [32, 33]. Further, sJIA is a heterogenous condition, and the role of IL-1 is less apparent in some patients [30, 34, 35]; indeed, Gattorno et al. identified two subsets of sJIA with distinct clinical features, based on (lack of) response to IL-1 blockade with anakinra. Taken together, it is possible that the lack of efficacy reported in some patients with sJIA in this study is related to lower IL-1 involvement in these patients. However, the low number of patients in the sJIA cohort limits the conclusions that can be drawn from our data.

The lack of efficacy in the FMF cohort of this study is also higher than reported in a systematic review of canakinumab in patients with colchicine-resistant FMF and those from the JIR cohort in France (8.6% vs.1.4% and 3.2%, respectively) [12, 26]. According to French reimbursement conditions, physicians must commit in writing to strictly adhere to EMA-approved indications. Additional restrictions include colchicine resistance (FMF) and severe disease (HIDS, TRAPS). Colchicine resistance criteria were not pre-defined in our study due to its observational nature; however, colchicine resistance is defined in the Belgian reimbursement criteria as having active disease with at least 1 crisis per month in the past year despite maximum tolerated dose, or documented intolerance despite preventive measures, per the colchicine SmPC [36]. As confirmation of *MEFV* mutations is not required for canakinumab reimbursement in Belgium and the collection of mutation data, although probably available, was not subject to this study, some patients were potentially misdiagnosed with FMF prior to study inclusion and would respond less well than anticipated to anti-IL-1 treatment. In 2 patients (1 FMF and 1 TRAPS), discontinuation of canakinumab was due to remission. It cannot be assumed that this was drug-free remission, as patients may have continued to receive treatments other than canakinumab during (and upon discontinuation of) the study. Moreover, drug-free remission is unexpected in these indications, particularly since the reimbursement criteria restrict canakinumab access to patients with severe disease activity only [28]. In the JIR cohort, 12.9% of patients with FMF and severe disease activity also discontinued canakinumab due to remission, but most patients were receiving concomitant colchicine [26]. Importantly, although centers provided the reason for canakinumab discontinuation, lack of efficacy and remission were defined per the investigators’ judgment and were therefore semi-subjective; this should be taken into consideration when drawing conclusions from these results.

Dosing and treatment intervals in this cohort were generally in line with the SmPC for patients with sJIA (for patients weighing ≥ 7.5 kg, 4 mg/kg, up to a maximum of 300 mg, every 4 weeks), while the maximum treatment intervals observed in patients with FMF, TRAPS, and MKD/HIDS were longer. Overall, 35 patients with FMF, TRAPS, or MKD/HIDS received ≥ 1 dose increase (≥ 150 mg) during the study. Adjustment of canakinumab dosing to optimize disease management was also observed in other real-world studies, including the RELIANCE and JIR registries of patients with FMF, TRAPS, and MKD/HIDS [25–27]. In the RELIANCE non-interventional study, patients with FMF and TRAPS typically received the RSD canakinumab at baseline, which progressed to a more even split between the three dosing categories (RSD, greater than RSD [> RSD], and less than RSD [< RSD]) at later timepoints. Most patients with MKD/HIDS received > RSD at baseline, which was maintained throughout the study [27]. In the JIR cohort, patients with FMF, TRAPS, and MKD/HIDS primarily received < RSD throughout the study, and treatment intervals varied greatly on a patient-by-patient basis, ranging from 6 to 10 weeks [25, 26].

No AEs, SAEs, ADRs, or SADRs were reported in the study, according to investigator reporting at the time of data collection; however, 10.4% patients discontinued canakinumab treatment due to a lack of efficacy during the observation period. On reconciliation of the study database following study completion, ‘lack of efficacy’ as a reason for discontinuation of canakinumab was retrospectively reported as an AE as part of Novartis’ pharmacovigilance protocol. There were no predefined criteria for lack of efficacy; these events were determined per investigators’ judgment.

Due to its observational nature, there are several limitations that must be considered before conclusions can be drawn from these findings. This non-interventional study was based on the collection of data from real-world clinical practice; as such, some variables were not captured, including patients’ medical history, prior and ongoing background treatment, disease severity (including complications, which may be expected due to the chronicity of illness), and genetic information. Similarly, no efficacy data relating to canakinumab treatment were collected, including laboratory analyses or other disease activity assessments; lack of efficacy was determined per the investigators’ judgment. Although the Belgian reimbursement criteria define treatment response, there were no further standardized response criteria provided to the treating physicians, which may limit the appropriate interpretation of the data here. Furthermore, data were missing for a number of patients throughout the study. Most notably, body weight was missing for most patients, meaning that the planned analysis of the evolution of dosing relative to that of body weight could not be completed. Eligible patients included those with sJIA, FMF, TRAPS, and MKD/HIDS; no patients with AOSD or CAPS were enrolled as these indications were not subject to the request from the Belgian health authorities. As such, no insights into canakinumab treatment patterns in these related conditions are available through this study. In addition, as sJIA, FMF, TRAPS, and MKD/HIDS are rare diseases, the patient populations enrolled in this study were small and study sites were selected based on membership in the Belgian Network for AIDs; recruitment may have been impacted by the potentially broad distribution of this relatively small number of eligible patients throughout Belgium. There are no publicly available Belgian data on the prevalence, incidence, or eligibility for biological treatment in these indications; the treating physician must propose registry inclusion for reimbursement, but the patient can refuse consent, so it is challenging to estimate the coverage of this registry, though we expect that it is high. The low patient numbers in the sJIA, TRAPS, and MKD/HIDS cohorts limit the conclusions that can be drawn from our results. In addition, the geographic diversity of this study is limited and may not be directly applicable to populations in other areas of the world. A higher proportion of patients aged < 18 years and with sJIA could have been expected based on the epidemiological data available in these diseases and the age at which they usually arise; eligibility may have been limited by the reimbursement criteria in these patients [37]. Finally, it is also possible that some eligible patients were not captured in the study as they (or their guardians) did not consent to participate. To overcome the limitations of our study, future research should be designed to collect more structured efficacy data, such as the inclusion of pre-defined response and lack of efficacy parameters, and ensure that genetic testing for FMF is carried out, to enable further analysis of the mutations present in these patients.

## Conclusions

Overall, the findings from this dataset (one of the largest available non-interventional studies of canakinumab in patients with sJIA, FMF, TRAPS, and MKD/HIDS) indicate that real-world treatment patterns for most patients in Belgium are generally aligned with the SmPC and the reimbursement criteria, and further demonstrate the well-tolerated safety profile of canakinumab. However, canakinumab access in eligible patients (per the SmPC and per international guidelines) may have been limited due to the Belgian reimbursement criteria, with a potential impact on the decisions made regarding the management of these patients. Closer alignment of the reimbursement criteria with recent disease management recommendations would ensure earlier access to effective treatment and subsequently improve disease outcomes in patients with sJIA, FMF, TRAPS, and MKD/HIDS in Belgium.

## Electronic supplementary material

Below is the link to the electronic supplementary material.

Supplementary Material 1

## Data Availability

All data relevant to the study are included in the article. Additional data are available from the corresponding author on reasonable request.

## Abbreviations

ACR: American College of Rheumatology
ADRs: Adverse drug reactions
AEs: Adverse events
AID: Autoinflammatory disease
AOSD: Adult-onset Still’s disease
CAPS: Cryopyrin-associated periodic syndromes
EULAR: European Alliance of Associations of Rheumatology
FMF: Familial Mediterranean fever
HIDS: Hyperimmunoglobulin-D syndrome
HPFS: Hereditary periodic fever syndromes
HRQoL: Health-related quality of life
IL: Interleukin
JIR: Juvenile Inflammatory Rheumatism
MKD: Mevalonate-kinase deficiency
NSAIDs: Non-steroidal anti-inflammatory drugs
PReS: Pediatric Rheumatology European Society
RIZIV/INAMI: Rijksinstituut voor ziekte- en invaliditeitsverzekering/Institut national d’assurance maladie-invalidité
RSD: Recommended starting dose
RWE: Real-world evidence
SADRs: Serious adverse drug reactions
SAEs: Serious adverse events
sJIA: Systemic juvenile idiopathic arthritis
SmPC: Summary of product characteristics
TRAPS: Tumor-necrosis factor receptor-associated periodic syndrome
