# Supplementary material for: Canakinumab treatment patterns in sJIA, FMF, TRAPS, and MKD/HIDS: real-world insights from a Belgian non-interventional study
Source: BMC Rheumatol. 2025 May 29;9:64. doi: 10.1186/s41927-025-00515-w (PMC12121185; doi:10.1186/s41927-025-00515-w)
Supplement: Supplementary file 1 — Supplementary Material 1 [file 41927_2025_515_MOESM1_ESM.docx]

# **Additional file 1**

**Canakinumab treatment patterns in sJIA, FMF, TRAPS, and MKD/HIDS:
real-world insights from a Belgian non-interventional study**

**Suppl. Table 1** Reimbursement criteria for canakinumab in Belgium [28]

| **Disease** | **Criteria for canakinumab initiation** | **Criteria for canakinumab continuation** |
| --- | --- | --- |
| **sJIA** | - Insufficient response to optimal NSAID use for 2–4 weeks or if NSAIDs are contraindicated - Preceding course of corticosteroids (1 mg/kg/day or 30 mg/day after 3 months, or 0.3 mg/kg/day or 10 mg/day after 6 months) - Insufficient response or intolerance to optimal use of tocilizumab | Patients must meet the defined response criteria after 6 months and every 12 months thereafter:   - ACR Pedi 30 response compared with the patient's clinical condition before treatment - Absence of fever associated with sJIA (no temperature ≥37.5°C in the previous 7 days) |
| **FMF, TRAPS or MKD/HIDS** | - FMF: 1 crisis per month over the past 12 months, despite maximum tolerated dose of colchicine, or documented intolerance despite preventative measures* - TRAPS or MKD/HIDS: 1 attack per month over the past 12 or 6 months, respectively - CRP ≥10 mg/l and - PGA score ≥2 | Patients must meet the defined response criteria after 16 weeks and every 12 months thereafter:   - CRP <10 mg/l and/or >70% reduction from baseline - PGA score <2 |

*Per colchicine SmPC [36]

ACR, American College of Rheumatology; CRP, C-reactive protein; FMF, familial Mediterranean fever; HIDS, hyperimmunoglobulinemia D syndrome; MKD, mevalonate kinase deficiency; NSAID, non-steroidal anti-inflammatory drug; PGA, physician’s global assessment; SmPC, summary of product characteristics; TRAPS, tumor necrosis factor receptor-associated periodic syndrome.
